# Long-term outcomes of ruptured hepatocellular carcinoma: international multicentre study

**DOI:** 10.1093/bjs/znae093

**Published:** 2024-04-17

**Authors:** Gaëtan-Romain Joliat, Robert de Man, Vincent Rijckborst, Matteo Cimino, Guido Torzilli, Gi Hong Choi, Hyung Soon Lee, Brian K P Goh, Takashi Kokudo, Chikara Shirata, Kiyoshi Hasegawa, Yujiro Nishioka, Jean-Nicolas Vauthey, Maria Baimas-George, Dionisios Vrochides, Nicolas Demartines, Nermin Halkic, Ismail Labgaa

**Affiliations:** Department of Visceral Surgery, Lausanne University Hospital CHUV, University of Lausanne (UNIL), Lausanne, Switzerland; Department of Gastroenterology and Hepatology, Erasmus Medical Centre, Rotterdam, the Netherlands; Department of Gastroenterology and Hepatology, Ikazia Hospital, Rotterdam, the Netherlands; Department of Hepatobiliary and General Surgery, Humanitas University, Humanitas Clinical and Research Centre, IRCCS, Rozzano, Milan, Italy; Department of Hepatobiliary and General Surgery, Humanitas University, Humanitas Clinical and Research Centre, IRCCS, Rozzano, Milan, Italy; Division of Hepatopancreatobiliary Surgery, Department of Surgery, Severance Hospital, Yonsei University College of Medicine, Seoul, Korea; Division of Hepatopancreatobiliary Surgery, Department of Surgery, Severance Hospital, Yonsei University College of Medicine, Seoul, Korea; Department of Hepatopancreatobiliary and Transplant Surgery, Singapore General Hospital, National Cancer Centre Singapore and Duke-National University of Singapore Medical School, Singapore, Singapore; Hepato-Biliary-Pancreatic Surgery Division, Department of Surgery, Graduate School of Medicine, University of Tokyo, Tokyo, Japan; Department of Visceral Surgery, Lausanne University Hospital CHUV, University of Lausanne (UNIL), Lausanne, Switzerland; Hepato-Biliary-Pancreatic Surgery Division, Department of Surgery, Graduate School of Medicine, University of Tokyo, Tokyo, Japan; Hepato-Biliary-Pancreatic Surgery Division, Department of Surgery, Graduate School of Medicine, University of Tokyo, Tokyo, Japan; Department of Surgical Oncology, University of Texas MD Anderson Cancer Center, Houston, Texas, USA; Department of Surgical Oncology, University of Texas MD Anderson Cancer Center, Houston, Texas, USA; Division of Hepatobiliary and Pancreatic Surgery, Department of Surgery, Carolinas Medical Centre, Charlotte, North Carolina, USA; Division of Hepatobiliary and Pancreatic Surgery, Department of Surgery, Carolinas Medical Centre, Charlotte, North Carolina, USA; Department of Visceral Surgery, Lausanne University Hospital CHUV, University of Lausanne (UNIL), Lausanne, Switzerland; Department of Visceral Surgery, Lausanne University Hospital CHUV, University of Lausanne (UNIL), Lausanne, Switzerland; Department of Visceral Surgery, Lausanne University Hospital CHUV, University of Lausanne (UNIL), Lausanne, Switzerland

## Introduction

Hepatocellular carcinoma (HCC) is one of the deadliest malignancies, with a cancer-related mortality ranking third after those for lung and colorectal cancer^1,2^. Spontaneous HCC rupture may occur in 5–10%^3^. Ruptured HCC can lead to haemorrhage and thereby result in high short-term mortality rates (30–70%)^4–6^. Although risk factors for rupture have been identified^7^, the underlying pathophysiology of ruptured HCC remains unknown. The evidence on ruptured HCC is limited, as it mainly derives from single-centre case series. Reports^8–14^ comparing outcomes of ruptured and non-ruptured HCC have demonstrated higher short-term mortality among patients with ruptured HCC, but data on long-term outcomes are scant. Available studies were essentially conducted in Asian cohorts. Studies including both Eastern and Western patients with ruptured HCC are lacking^3,9^. Moreover, patients with non-ruptured and ruptured HCC in these studies were not necessarily comparable.

The present large-scale multicentre study aimed to characterize patients with ruptured HCC and compare long-term outcomes after surgery with those of patients who underwent resection of non-ruptured HCC, using propensity score matching (PSM).

## Methods

This was a multicentre, retrospective study of consecutive patients with ruptured HCC receiving any type of treatment and patients with non-ruptured HCC after partial hepatectomy between 1 January 2000 and 31 December 2017. Only spontaneous ruptures were considered. Rupture was defined as a breach of the hepatic capsule with or without haemorrhage. Patients with and without ruptured HCC were compared using PSM. Matching criteria were defined *a priori*, and included age, preoperative α-fetoprotein level, tumour size on imaging, presence of cirrhosis, Child–Pugh grade, Barcelona Clinic Liver Cancer classification, resection status, tumour grade, and microvascular invasion. Details of methods are available in the *Supplementary material*.

The study protocol was reviewed and approved by the ‘Commission cantonale d’éthique de la recherche’, Lausanne, Switzerland (approval number 2019-00314) (leading site ethics commission).

## Results

### Patients

Of 2033 patients included, 226 had a ruptured HCC and 1807 a non-ruptured HCC. The number of patients per institution can be found in the *Supplementary material*. Patient characteristics are summarized in *Table S1*.

With a median follow-up of 71 (95% c.i. 56 to 86) months, disease-free survival (DFS) and overall survival (OS) for the entire HCC cohort were 48 (95% c.i. 43 to 53) and 54 (50 to 58) months, respectively. In multivariable analysis, factors associated with rupture were preoperative albumin level (OR 1.23, 95% c.i. 1.11 to 1.32; *P* = 0.001), Model for End-Stage Liver Disease (MELD) score (OR 1.08, 1.04 to 1.12; *P* = 0.003), ASA grade (OR 3.53, 1.64 to 7.61; *P* = 0.002), and Child–Pugh grade (OR 43.12, 1.52 to 1200.54; *P* = 0.027).

### Patients with ruptured hepatocellular carcinoma

Patients with ruptured HCC were managed with upfront surgery (68, 30.1%), surgery after embolization (104, 46.0%) (*Table S2*), transarterial chemoembolization (46, 20.4%) or best supportive care (8, 3.5%).

Median DFS and OS among all patients with ruptured HCC were 10 (95% c.i. 7 to 13) and 21 (12 to 30) months, respectively. In multivariable regression analysis, Child–Pugh grade A (HR 2.23, 95% c.i. 1.21 to 3.84; *P* = 0.008), R0 resection (HR 2.24, 1.23 to 4.04; *P* = 0.008), and preoperative embolization (HR 2.59, 1.52 to 4.58; *P* < 0.001) were independently associated with longer OS (*Table 1*). The 172 patients who underwent resection had longer median OS than the 54 patients who did not undergo surgery (32 (19 to 45) *versus* 10 (6 to 14) months respectively; *P* < 0.001) (*Fig. S1*). Corresponding median progression-free survival was 11 (7 to 15) *versus* 10 (6 to 14) months (*P* = 0.008). Among the 226 patients with ruptured HCC, recurrence was observed in 115 (50.9%), including 61 intrahepatic recurrences (53.0%), 19 extrahepatic recurrences (16.5%), and 35 mixed patterns of recurrence (30.4%). Peritoneal recurrence was found in 12 patients, meaning that 7.0% of patients with resected ruptured HCC (12 of 172) developed peritoneal implants. OS was longer in patients with delayed *versus* upfront surgery (76 (38 to 114) *versus* 20 (13 to 27) months; *P* < 0.001).

**Table 1:** Univariable and multivariable Cox regression analyses of prognostic factors for overall survival in the entire cohort of 226 patients with ruptured hepatocellular carcinoma

	Univariable analysis		Multivariable analysis	
	HR	*P*	HR	*P*
Age (years)	1.00 (1.00, 1.00)	0.730		
Female sex	0.92 (0.61, 1.41)	0.684		
ASA grade I–II	1.24 (0.82, 1.73)	0.313		
BMI (kg/m^2^)	1.01 (0.99, 1.04)	0.184		
Cirrhosis	1.44 (1.03, 2.04)	0.030	1.43 (0.92, 2.38)	0.155
Child grade A	1.62 (1.11, 2.43)	0.015	2.21 (1.21, 3.84)	0.008
BCLC stage 0–A	1.29 (0.92, 1.86)	0.208		
NASH	0.81 (0.42, 1.43)	0.409		
Diabetes mellitus	0.94 (0.63, 1.31)	0.506		
MELD score	1.01 (1.00, 1.02)	0.743		
AFP (ng/ml)	1.00 (1.00, 1.00)	0.027	1.00 (1.00, 1.00)	0.356
Largest nodule on CT (mm)	1.02 (1.01, 1.03)	0.151		
No. of nodules	0.93 (0.67, 1.14)	0.404		
Tumour grade G1–G2	1.42 (0.93, 2.21)	0.090	1.43 (0.84, 2.33)	0.230
Microvascular invasion	1.83 (1.18, 2.76)	0.007	1.32 (0.73, 2.34)	0.374
R0 resection*	0.32 (0.22, 0.43)	< 0.001	2.21 (1.24, 4.01)	0.008
Embolization	0.83 (0.52, 1.11)	0.100	2.64 (1.53, 4.64)	< 0.001

HRs for continuous variables are shown per unit increase. Values in parentheses are 95% confidence intervals. *At admission or as a second-step procedure. BCLC, Barcelona Clinic Liver Cancer; NASH, non-alcoholic steatohepatitis; MELD, Model for End-Stage Liver Disease; AFP, α-fetoprotein.

### Comparison of outcomes of patients with ruptured *versus* non-ruptured tumours

*Table S3* summarizes the characteristics of adjusted groups after PSM. There was no difference in postoperative complication rate between patients who underwent resection for ruptured *versus* non-ruptured HCC (50.0 *versus* 37.7%; *P* = 0.072). Median Comprehensive Complication Index scores were no different between groups (20.9 *versus* 0; *P* = 0.092). Corresponding 90-day mortality rates were 7.5 *versus* 2.8% (*P* = 0.122).

**Fig. 1:**
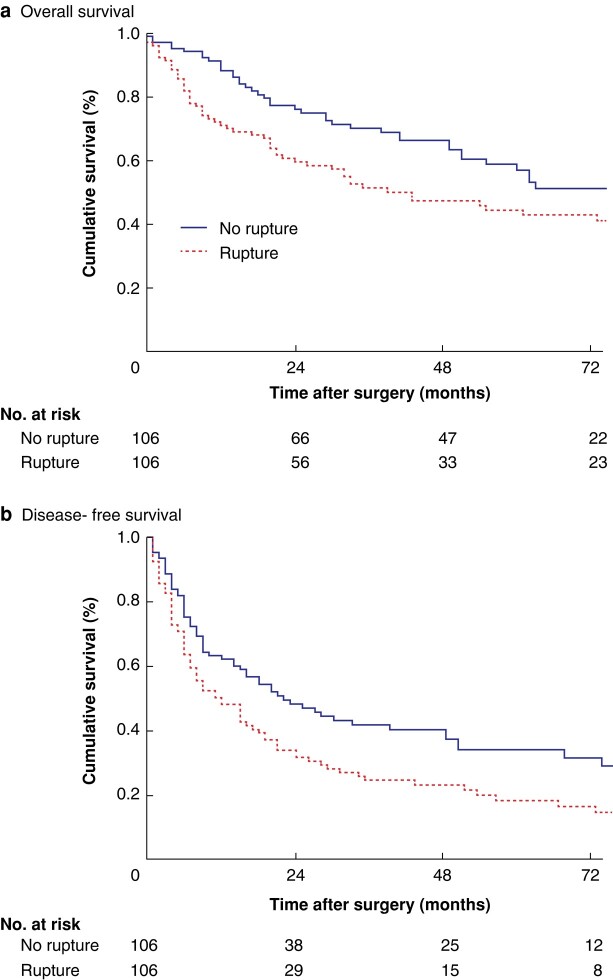
Kaplan–Meier curves showing overall and disease-free survival of patients with ruptured *versus* non-ruptured hepatocellular carcinoma after propensity score matching **a** Overall and **b** disease-free survival. **a**  *P* = 0.014, **b**  *P* = 0.011 (log rank test).

Patients with ruptured HCC had worse median OS (43 (95% c.i. 21 to 65) *versus* 100 60 to 140) months; *P* = 0.014, log rank test) (*Fig. 1a*) and DFS (12 (7 to 17) *versus* 22 (12 to 32) months; *P* = 0.011) (*Fig. 1b*) than those with non-ruptured HCC.

Corresponding recurrence rates were 74.2 and 51.3% (*P* < 0.001). Intrahepatic and extrahepatic recurrence rates did not differ between groups, but mixed intrahepatic and extrahepatic patterns of recurrence were more common in patients with ruptured HCC (25.4 *versus* 4.3%; *P* < 0.001). The peritoneal recurrence rate did not differ between patients who underwent resection for ruptured *versus* non-ruptured HCC (8.1 *versus* 5.4%; *P* = 0.269).

## Discussion

Factors associated with risk of ruptured HCC were preoperative albumin level, MELD score, ASA grade, and Child–Pugh grade. DFS and OS were worse in patients with ruptured HCC. Child A grade, arterial embolization, and complete resection were independent prognostic factors for longer OS in patients with ruptured HCC.

Median OS for patients with ruptured HCC was longer in the present study than in a Japanese nationwide analysis^4^ including 1160 patients with ruptured HCC (228 (95% c.i. 196 to 273) days. Looking at operated patients, the 5-year OS rate was 33.9% in the study by Aoki *et al.*^4^ and liver resection was the treatment modality associated with best survival. Similar findings regarding DFS and OS were reported in a retrospective, single-centre study^15^ from China including 143 patients after partial hepatectomy. Five-year OS and DFS were shorter in patients with ruptured *versus* non-ruptured HCC (16.8 *versus* 50.5%, *P* < 0.001; 14.8 *versus* 43.7%, *P* < 0.001)^15^. Patients with ruptured HCC and upfront hepatectomy had lower OS and DFS rates than those who underwent hepatectomy after embolization^15^. Albeit multicentre or large, these studies had a nationwide or single-centre design and included no Western patients, which may preclude extrapolation of their results and conclusions. Conversely, a recent single-centre PSM study^16^ noted that patients with ruptured HCC had similar OS to those with non-ruptured HCC after surgery.

In contrast to previous reports^7^, tumour size was not identified as independent prognostic factor in the present analysis. The prognostic factors identified in this study are important findings that need to be interpreted with caution and deserve confirmation with prospective data. The present findings, together with other data from the literature, strongly suggest that embolization should be undertaken before surgery in patients with ruptured HCC, whenever possible. Preoperative embolization has several advantages, such as patient resuscitation and avoidance of emergency surgery. This strategy still offers patients the option of proceeding to surgical resection, which is the main curative option in HCC.

Theoretically, a high rate of peritoneal recurrences may be anticipated in patients undergoing resection of ruptured HCC. This was limited to 7.0% of patients in the present cohort, concordant with other studies^11,17^.

Spontaneous rupture is unlikely to be a random event, but rather a consequence of the biological traits of the tumour. An interesting study by Nault *et al.*^18^ showed that specific subtypes of hepatocellular adenoma had an increased risk of bleeding (β-catenin and sonic hedgehog activation) or malignant transformation. A parallel hypothesis can be made for ruptured HCC, particularly when noting that patients with rupture were more often women. It would be interesting to obtain molecular data and investigate the role of hormones in the event of ruptured HCC. Molecular analyses may help in subclassifying ruptured HCC to better tailor its therapeutic management^19^.

## Supplementary Material

znae093_Supplementary_Data

## Data Availability

Research data supporting this publication are available directly from the corresponding authors on reasonable request.
