# Supplementary material for: Long-term outcomes of ruptured hepatocellular carcinoma: international multicentre study
Source: Br J Surg. 2024 Apr 17;111(4):znae093. doi: 10.1093/bjs/znae093 (PMC11023170; doi:10.1093/bjs/znae093)
Supplement: znae093_Supplementary_Data [file znae093_supplementary_data.docx]

**Long-term Outcomes of Ruptured Hepatocellular Carcinoma: An International Multicentre Study**

Gaëtan-Romain Joliat, MD^1*^, Robert de Man, MD, PhD^2^, Vincent Rijckborst, MD, PhD^3^, Matteo Cimino, MD^4^, Guido Torzilli, MD, PhD^4^, Gi Hong Choi, MD^5^, Hyung Soon Lee, MD^5^, Brian K. P. Goh, MBBS, MMed, MSc^6^, Takashi Kokudo, MD, PhD^7^, Chikara Shirata, MD^1,7^, Kiyoshi Hasegawa, MD, PhD^7^, Yujiro Nishioka, MD, PhD^8^, Jean-Nicolas Vauthey, MD^8^, Maria Baimas-George, MD, MPH^9^, Dionisios Vrochides, MD, PhD^9^, Nicolas Demartines, MD^1*^, Nermin Halkic, MD^1^, Ismail Labgaa, MD, PhD^1*^

^1^ Department of Visceral Surgery, Lausanne University Hospital CHUV, University of Lausanne (UNIL), Lausanne, Switzerland.

^2^ Department of Gastroenterology and Hepatology, Erasmus Medical Center, Rotterdam, The Netherlands.

^3^ Department of Gastroenterology and Hepatology, Ikazia Hospital, Rotterdam, The Netherlands.

^4^ Department of Hepatobiliary and General Surgery, Humanitas University, Humanitas Clinical and Research Center, IRCCS, Rozzano, Milan, Italy.

^5^ Division of Hepatopancreatobiliary Surgery, Department of Surgery, Severance Hospital, Yonsei University College of Medicine, Seoul, Korea.

^6^ Department of Hepatopancreatobiliary and Transplant Surgery, Singapore General Hospital, National Cancer Center Singapore and Duke-National University of Singapore Medical School, Singapore, Singapore.

^7^ Hepato-Biliary-Pancreatic Surgery Division, Department of Surgery, Graduate School of Medicine, The University of Tokyo, Tokyo, Japan.

^8^ Department of Surgical Oncology, The University of Texas MD Anderson Cancer Center, Houston, Texas, USA.

^9^ Division of Hepatobiliary and Pancreatic Surgery, Department of Surgery, Carolinas Medical Center, Charlotte, North Carolina, USA.

*** Correspondence and requests for reprints to:**

Gaëtan-Romain Joliat, MD

Department of Visceral Surgery

Lausanne University Hospital CHUV

Rue du Bugnon 46

1011 Lausanne, Switzerland

Phone: +41 79 556 42 93

Email: gaetan.joliat@gmail.com

Nicolas Demartines, MD, FACS, FRCS

Prof. and Chairman

Department of Visceral Surgery

Lausanne University Hospital CHUV

Rue du Bugnon 46

1011 Lausanne, Switzerland

Phone: +41 21 314 24 00

Email: demartines@chuv.ch

Ismail Labgaa, MD, PhD

Department of Visceral Surgery

Lausanne University Hospital CHUV

Rue du Bugnon 46

1011 Lausanne, Switzerland

Phone: +41 79 556 68 47

Email: ismail.labgaa@chuv.ch

**Supplementary Materials - Index**

| **Supplementary Methods** |  |
| --- | --- |
| Study design and patients | *page 4* |
| Management of rupture  Propensity score matching (PSM)  Statistics | *page 5*  *page 5*  *page 6* |
| **Supplementary Results** | *page 7* |
| **Supplementary Discussion** | *page 8* |
| **Supplementary Figures and Tables** |  |
| Figure S1 | *page 9* |
| Table S1  Table S2  Table S3 | *page 10*  *page 12*  *page 13* |
| **References** | *page 14* |
|  |  |

**Supplementary Methods**

*Study design and patients*

This cross-sectional study included HCC patients from 8 academic centers in US, Europe, and Asia: Lausanne University Hospital CHUV (Lausanne, Switzerland), Erasmus Medical Center (Rotterdam, The Netherlands), Humanitas University (Milan, Italy), Severance Hospital (Seoul, Korea), Singapore General Hospital (Singapore, Singapore), The University of Tokyo Hospital (Tokyo, Japan), The University of Texas MD Anderson Cancer Center (Houston, USA), and Carolinas Medical Center (Charlotte, USA). Prospectively maintained or collected databases of these centers were retrospectively analyzed^1–4^. Consecutive rHCC patients receiving any type of treatment and nrHCC patients after partial hepatectomy between January 1, 2000 and December 31, 2017 were included. Diagnosis of HCC was based on histological analyses of the resected specimen in surgical patients and on imaging criteria according to the Liver Reporting and Data System (Li-RADS®) of the American College of Radiology, in rHCC patients receiving non-surgical therapies^5^. Patients <18 years, without informed consent, with HCC recurrence or with mixed HCC-cholangiocarcinoma were excluded.

Only spontaneous ruptures were considered. Rupture was defined as a breach of the hepatic capsule with or without hemorrhage^6^. The diagnosis of rHCC was based on contrast-enhanced abdominal computed tomography (CT). Typical radiological findings on CT were HCC with focal discontinuity of the liver surface, subcapsular hematoma, or active contrast product extravasation.

Complete histological resection (R0) was defined as the absence of tumoral cells within 1 mm of the surgical margin on histology. Postoperative complications were graded according to Clavien classification^7^ and the Comprehensive Complication Index (CCI) was calculated for each patient^8^. HCC recurrence was defined as new occurrence of hepatic or extrahepatic HCC after R0 resection and/or disease progression after R1 resection.

The primary endpoints of the study were disease-free survival (DFS) and overall survival (OS) calculated from the day of surgery in operated patients and from the day of rupture in non-operated rHCC patients.

This manuscript followed the Strengthening the Reporting of Observational Studies in Epidemiology (STROBE) guidelines^9^ and the study protocol was approved by the institutional review boards of each participating center.

*Management of rupture*

HCC rupture was managed according to the policy and guidelines of each participating center^10,11^. It included either surveillance, upfront surgery, or embolization. Upfront surgery or embolization were typically performed in case of hemorrhagic shock or active bleeding detected on imaging. Patients who were not operated upfront had a delayed hepatectomy (staged hepatectomy several days or weeks after rHCC occurrence), systemic therapy or transarterial chemotherapy, or palliative care.

*Propensity score matching (PSM)*

Criteria of matching were defined *a priori*. These criteria were determined based on evidence from the available literature on potential confounding and prognostic factors of survival in HCC^12^. Of importance, they did not derive from potentially detected statistical differences between rHCC and nrHCC groups. This list of factors included: age, preoperative alpha-fetoprotein (AFP) level, tumor size on imaging, presence of cirrhosis, Child-Pugh score, Barcelona Clinic Liver Cancer (BCLC) classification, resection status, tumor grade, and microvascular invasion. Propensity score was calculated using a logistic regression model, and 1:1 matching was performed with the nearest neighbor method and a maximal caliper width of 0.2 standard deviation^13^. Differences between rHCC and nrHCC groups were assessed before and after matching. Of note, the BCLC classification does not take into account the occurrence of rupture.

*Statistics*

Summary of continuous and categorical data were expressed in median with interquartile range (IQR) and in number with percentage, respectively. Comparisons between continuous variables were made using Student t-tests. Categorical variables were compared with chi-square tests. Survival analyses were performed with Kaplan-Meier curves and comparisons were made using the log-rank test. Events for DFS were defined as recurrence or death. Median follow-up was calculated using the inverse Kaplan-Meier method. Predictive factors of survival were calculated using a Cox proportional hazards regression model and their effect was reported as hazard ratio (HR) with 95% confidence interval (CI). Risk factors of rupture were calculated using a binary logistic regression and were reported as odds ratio (OR) with 95% CI. A p-value <0.05 was considered significant. All p-values were two-sided (two-tailed).

Statistics were performed using SPSS 28 and STATA 17 for Mac OS X.

**Supplementary Results**

The number of included rHCC and nrHCC patients per institution was as follows:

- Lausanne University Hospital CHUV (Lausanne, Switzerland): rHCC, n=16; nrHCC, n=159
- Erasmus Medical Center (Rotterdam, The Netherlands): rHCC, n=11
- Humanitas University (Milan, Italy): rHCC, n=9; nrHCC, n=254
- Severance Hospital (Seoul, Korea): rHCC, n=73; nrHCC, n=1032
- Singapore General Hospital (Singapore, Singapore): rHCC, n=78
- The University of Tokyo Hospital (Tokyo, Japan): rHCC, n=23
- The University of Texas MD Anderson Cancer Center (Houston, USA): rHCC, n=6; nrHCC, n=245
- Carolinas Medical Center (Charlotte, USA): rHCC, n=10; nrHCC, n=117

**Supplementary Discussion**

Several limitations of this cross-sectional study have to be mentioned. The retrospective design may allow data collection or selection biases. Another limitation is the heterogeneity of the patients due to the specific rHCC management of each participating center. The choice of matching criteria was defined beforehand (a priori) and was based on prognostic factors of survival. Choosing other matching criteria such as resection type (minor/major hepatectomy) might have influenced the matched cohort. Finally, this study focused on clinical data and lacked molecular analyses.

On the other hand, the strengths of the present study are its multicentric and international design, a unique feature allowing to include a large number of patients giving a robust statistical power. For this reason, collected patient reflected the whole spectrum of HCC, with various underlying liver diseases. Moreover, this study included data from Western and Eastern countries, picturing the global clinical landscape of rHCC. The use of a PSM approach permitted to conduct adjusted comparisons between rHCC and nrHCC patients. PSM took into account potential confounding factors, an unprecedented effort on rHCC in Western and Eastern countries, that permitted to better understand and delineate this disease.

**Supplementary Figures and Tables**

**Figure S1**

**
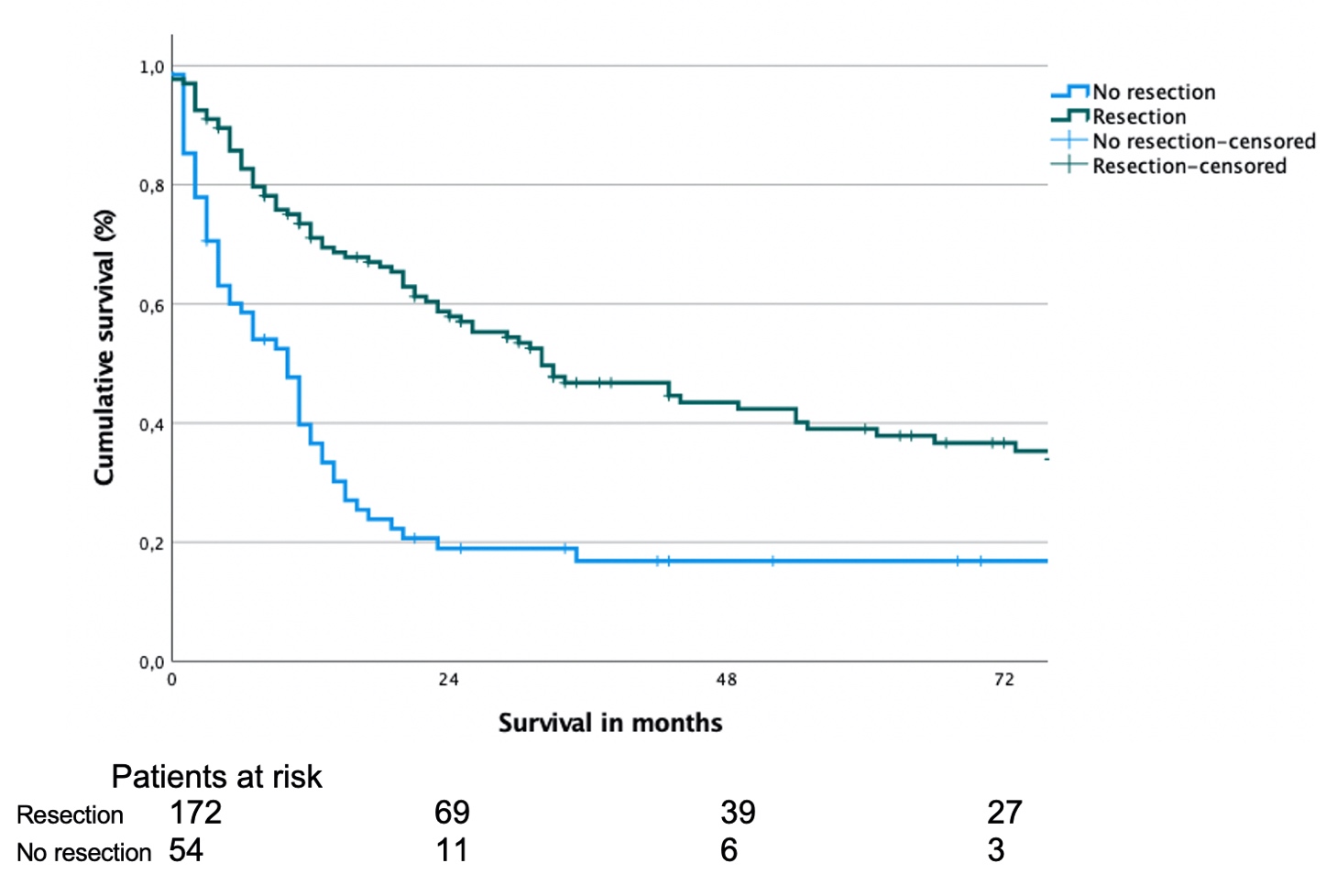
**

**Table S1.** Characteristics of unadjusted patients with ruptured HCC (rHCC) and with non-ruptured HCC (nrHCC).

|  | rHCC  (n=226) | nrHCC  (n=1807) | p-value |
| --- | --- | --- | --- |
| Age, *years* | 63 (54-72) | 62 (55-70) | 0.186 |
| Gender, *women* | 41 (18%) | 210 (12%) | **0.005** |
| ASA scores I-II | 142 (63%) | 587 (32%) | **<0.001** |
| BMI, *kg/m^2^* | 24.2 (22.0-27.6) | 24.2 (22.1-27.0) | 0.918 |
| Underlying liver diseases  Viral hepatitis  Alcohol hepatitis  NASH  Others  Unknown | 97 (43%)  13 (6%)  38 (17%)  49 (22%)  29 (12%) | 930 (51%)  84 (5%)  436 (24%)  331 (18%)  26 (2%) | **0.015**  0.463  **0.014**  0.221  **<0.001** |
| Cirrhosis | 108 (48%) | 506 (28%) | **<0.001** |
| Child score B-C | 50 (22%) | 19 (1%) | **<0.001** |
| BCLC stage 0-A | 64 (28%) | 1336 (74%) | **<0.001** |
| Diabetes mellitus | 46 (20%) | 240 (13%) | **<0.001** |
| MELD score | 9 (8-11) | 7 (7-9) | **0.001** |
| AFP level, *ng/ml* | 103 (7-1424) | 12 (4-119) | **<0.001** |
| Largest nodule on CT, *mm* | 80 (58-100) | 35 (22-55) | **<0.001** |
| Number of nodules | 1 (1-1) | 1 (1-1) | 0.642 |
| Resection status R0* | 128 (57%) | 1627 (90%) | **<0.001** |
| Tumor grade G1-G2 | 87 (38%) | 1301 (72%) | **<0.001** |
| Microvascular invasion | 63 (28%) | 733 (41%) | **<0.001** |

Data are shown as median with interquartile range or number with percentage. Significant p-values appear in bold.

ASA: American Society of Anesthesiologists, BMI: body-mass index, NASH: non alcoholic steatohepatitis, BCLC: Barcelona Clinic Liver Cancer, MELD: Model for End-Stage Liver Disease, AFP: alpha fetoprotein, CT: computed tomography.

* The rest of the patients had R1 resections as no patient had an R2 resection.

**Table S2.** Summary of operative treatments and short-term outcomes of patients with ruptured hepatocellular carcinoma (n=172).

|  | Upfront surgery  (n=68) | Surgery after embolization  (n=104) |
| --- | --- | --- |
| Microwave ablation | 1 (1%) | 0 |
| Partial hepatectomy | 67 (99%) | 104 (100%) |
| Minor/major resections | 39 (57%)/28 (43%) | 64 (62%)/40 (38%) |
| Intraoperative blood loss, *mL* | 700 (400-1200) | 725 (350-1200) |
| Intraoperative transfusion | 30 (44%) | 34 (33%) |
| Median transfusion volume, *mL* | 710 (400-1800) | 550 (338-1088) |
| Operative time, *min* | 180 (135-265) | 264 (195-384) |
| 90-day mortality | 9 (13%) | 1 (1%) |
| Length of stay, *days* | 8 (6-11) | 20 (10-34) |

Number with percentage or median with interquartile rate.

**Table S3.** Characteristics of patients with ruptured HCC (rHCC) and non-ruptured HCC (nrHCC) adjusted after propensity score matching.

|  | rHCC  n=106 | nrHCC  n=106 | p-value |
| --- | --- | --- | --- |
| Age, *years* | 64 (56-73) | 68 (60-75) | 0.063 |
| Gender, *women* | 19 (18%) | 22 (21%) | 0.602 |
| ASA score I/II-III | 23 (22%)/83 (78%) | 18 (17%)/88 (83%) | 0.385 |
| BMI, *kg/m^2^* | 24 (22-27) | 24 (22-28) | 0.260 |
| Cirrhosis | 56 (53%) | 65 (61%) | 0.212 |
| Child score B-C | 6 (6%) | 7 (7%) | 0.775 |
| BCLC stage 0-A | 31 (29%) | 44 (42%) | 0.062 |
| NASH | 12 (11%) | 9 (8%) | 0.490 |
| Diabetes mellitus | 24 (22%) | 17 (16%) | 0.224 |
| MELD score | 9 (7-10) | 8 (7-11) | 0.420 |
| AFP, *ng/mL* | 136 (7-1748) | 17 (3-160) | 0.163 |
| Largest nodule on CT, *mm* | 80 (57-101) | 71 (45-120) | 0.430 |
| Number of nodules | 1 (1-1) | 1 (1-2) | 0.089 |
| Resection status R0* | 84 (79%) | 90 (85%) | 0.283 |
| Tumor grade G1-G2 | 52 (49%) | 50 (47%) | 0.783 |
| Microvascular invasion | 42 (40%) | 46 (43%) | 0.577 |

Data are shown as median with interquartile range or number with percentage.

ASA: American Society of Anesthesiologists, BMI: body-mass index, BCLC: Barcelona Clinic Liver Cancer, NASH: non alcoholic steatohepatitis, MELD: Model for End-Stage Liver Disease, AFP: alpha fetoprotein, CT: computed tomography.

* The rest of the patients had R1 resections as no patient had an R2 resection.

**References**

1 Joliat G-R, Labgaa I, Uldry E, Demartines N, Halkic N. Recurrence rate and overall survival of operated ruptured hepatocellular carcinomas. *Eur J Gastroenterol Hepatol*. 2018; **30**: 792–796.

2 Rijckborst V, Ter Borg MJ, Tjwa ET, Sprengers D, Verhoef K, Moelker A, *et al.* Short article: Management of ruptured hepatocellular carcinoma in a European tertiary care center. *Eur J Gastroenterol Hepatol*. 2016 Aug; **28**: 963–966.

3 Lee HS, Choi GH, Kang DR, Han K-H, Ahn SH, Kim DY, *et al.* Impact of spontaneous hepatocellular carcinoma rupture on recurrence pattern and long-term surgical outcomes after partial hepatectomy. *World J Surg*. 2014 Aug; **38**: 2070–2078.

4 Chua DW, Koh Y-X, Allen JC, Chan C-Y, Lee S-Y, Cheow P-C, *et al.* Impact of spontaneous rupture on the survival outcomes after liver resection for hepatocellular carcinoma: A propensity matched analysis comparing ruptured versus non-ruptured tumors. *Eur J Surg Oncol*. 2019 Sep; **45**: 1652–1659.

5 Chapter-8-LIRADS-Categories.pdf [Internet]. [cited 2022 Sep 20]. Available from: https://www.acr.org/-/media/ACR/Files/Clinical-Resources/LIRADS/Chapter-8-LIRADS-Categories.pdf?la=en&hash=6008F7A3B8E2475E4C1DC2C8974A052D

6 Yang T, Sun YF, Zhang J, Lau WY, Lai ECH, Lu JH, *et al.* Partial hepatectomy for ruptured hepatocellular carcinoma. *Br J Surg*. 2013 Jul; **100**: 1071–1079.

7 Dindo D, Demartines N, Clavien P-A. Classification of surgical complications: a new proposal with evaluation in a cohort of 6336 patients and results of a survey. *Ann Surg*. 2004 Aug; **240**: 205–213.

8 Slankamenac K, Graf R, Barkun J, Puhan MA, Clavien P-A. The comprehensive complication index: a novel continuous scale to measure surgical morbidity. *Ann Surg*. 2013 Jul; **258**: 1–7.

9 von Elm E, Altman DG, Egger M, Pocock SJ, Gøtzsche PC, Vandenbroucke JP, *et al.* The Strengthening the Reporting of Observational Studies in Epidemiology (STROBE) Statement: guidelines for reporting observational studies. *Int J Surg*. 2014 Dec; **12**: 1495–1499.

10 Aoki T, Kokudo N, Matsuyama Y, Izumi N, Ichida T, Kudo M, *et al.* Prognostic impact of spontaneous tumor rupture in patients with hepatocellular carcinoma: an analysis of 1160 cases from a nationwide survey. *Ann Surg*. 2014 Mar; **259**: 532–542.

11 Yang T, Sun YF, Zhang J, Lau WY, Lai ECH, Lu JH, *et al.* Partial hepatectomy for ruptured hepatocellular carcinoma. *Br J Surg*. 2013 Jul; **100**: 1071–1079.

12 Reig M, Forner A, Rimola J, Ferrer-Fàbrega J, Burrel M, Garcia-Criado Á, *et al.* BCLC strategy for prognosis prediction and treatment recommendation: The 2022 update. *J Hepatol*. 2022 Mar; **76**: 681–693.

13 Austin PC. Optimal caliper widths for propensity-score matching when estimating differences in means and differences in proportions in observational studies. *Pharm Stat*. 2011 Apr; **10**: 150–161.
